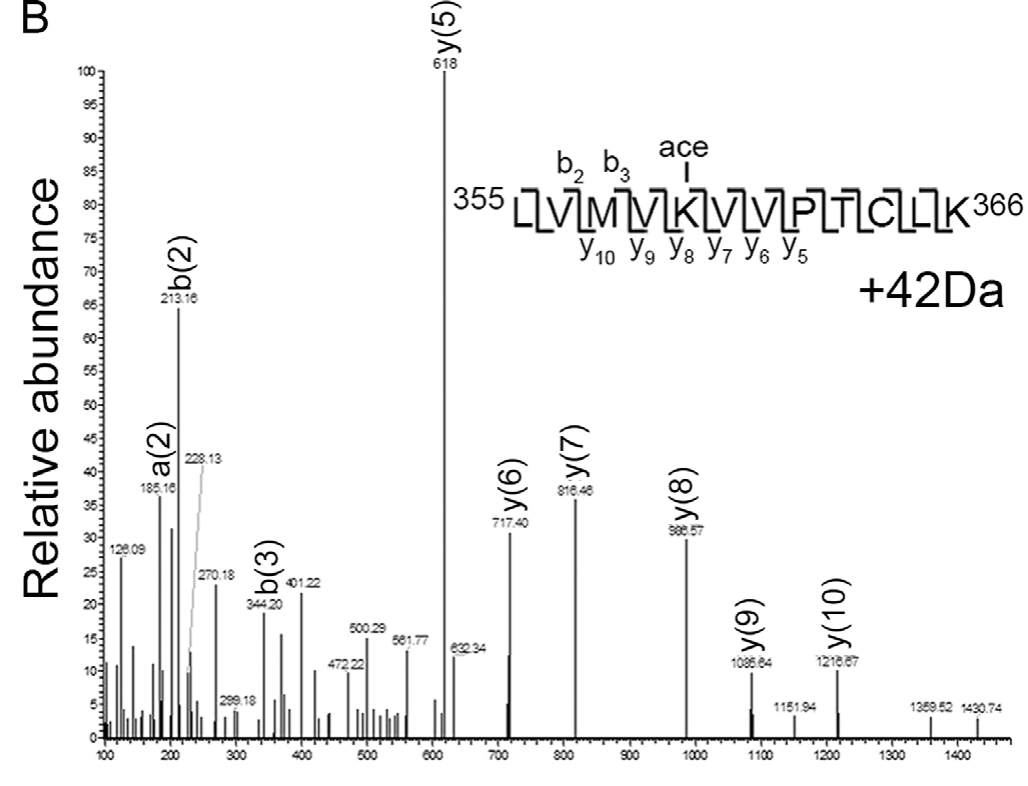# Correction: KAT8 selectively inhibits antiviral immunity by acetylating IRF3

**DOI:** 10.1084/jem.2018177303182019c

**Published:** 2019-03-21

**Authors:** Wanwan Huai, Xingguang Liu, Chunmei Wang, Yunkai Zhang, Xi Chen, Xiang Chen, Sheng Xu, Tim Thomas, Nan Li, Xuetao Cao

Vol. 216, No. 4, April 1, 2019. 10.1084/jem.20181773.

The authors regret that in the original version of this paper, the peptide sequence in Fig. 6 B appeared incorrectly as “LVMVKVVP.” Panel B appears below with the corrected peptide sequence, “LVMVKVVPTCLK.” All versions of this article have been corrected.

**Figure:**